# A Rare Case of Statin-Induced Rhabdomyolysis With Severe Acute Kidney Injury After a Decade of Therapy

**DOI:** 10.7759/cureus.72913

**Published:** 2024-11-03

**Authors:** Oghenevwede A Okuma, Nikitha Shetty, Priyanka Kapur, Christabel Ovesuor, Courage Idahor

**Affiliations:** 1 Internal Medicine, County Durham and Darlington Foundation Trust, Darlington, GBR; 2 General Internal Medicine, County Durham and Darlington Foundation Trust, Darlington, GBR; 3 Internal Medicine, Federal Medical Centre (FMC), Asaba, NGA; 4 Emergency Medicine, Barking, Harvering and Redbridge Foundation Trust, London, GBR

**Keywords:** acute kidney injury, prolomged statin induced rhabdomyolysis, rhabdomyolysis, severe aki, statin induced

## Abstract

Cardiovascular diseases are a major global health concern, with statin therapy playing a significant role in primary and secondary prevention. Statin-associated muscle symptoms typically occur early in treatment, but severe rhabdomyolysis is a rare complication. We present an unprecedented case of a patient who developed severe rhabdomyolysis with acute kidney injury after 10 years of uninterrupted statin therapy. The 10 years of therapy in this case is the longest duration reported in the literature. While several risk factors are associated with its development, including alcohol abuse, our patient's case stands out due to the extended latency of 10 years between statin initiation and rhabdomyolysis onset. Chronic alcohol consumption compounded the diagnostic challenge by masking the symptoms. The pathogenesis involves both muscle toxicity and mitochondrial dysfunction. Timely diagnosis and discontinuation of statin therapy, aggressive hydration, and vigilant monitoring are crucial for managing this life-threatening condition.

## Introduction

Cardiovascular events, including stroke and acute coronary syndromes, are a leading global cause of morbidity and mortality. These events contributed to 32% of all recorded deaths worldwide in 2019 [1]. Among the strategies developed to reduce the burden and risk of cardiovascular events, statin therapy in primary and secondary prevention has shown considerable benefits and forms a key part of recommendations from professional bodies, such as the American Society of Cardiology, the European Society of Cardiology, and the UK National Institute for Health and Care Excellence [2]. Data from over 30 years of statin use suggest they are safe and that serious adverse effects are rare. The risk of rhabdomyolysis is reported to be approximately 1/10,000 person-years of treatment. The incidence of severe rhabdomyolysis is even rare [3]. Statin-associated muscle symptoms usually occur early, following the commencement of treatment [3]. It is rare for patients to develop rhabdomyolysis after years of tolerating therapy [3,4]. Here, we present the case of a patient who developed severe rhabdomyolysis after approximately 10 years of tolerating therapy without a change in dose or change in medication. Ten years of therapy is the second longest duration recorded in the literature at the time of writing this report. One case report noted evidence of rhabdomyolysis after 20 years of statin therapy, which is the longest reported so far in the literature [5]. Such a delayed onset of this adverse event is novel, with most studies reporting effects within weeks or months. A high index of suspicion is therefore required.

## Case presentation

Our patient, a man in his 60s, attended the hospital with a two-week history of progressively worsening fatigue with muscle pain, muscle weakness, and reduced mobility associated with low urine output, brown-colored urine, and jaundice. The latter symptom was noticed by the patient’s family. There was no history of diarrhea or vomiting. He had an ischaemic stroke 10 years ago and has since been on Aspirin 75 mg once daily, Atorvastatin 40 mg once daily, and Indapamide 2.5 mg once daily. Apart from hypertension, there is no other medical history and no recent change in medication. He has tolerated all medications at the current dose for 10 years. He reported drinking an average of half a bottle of whisky every day and had done so for many years, but had never smoked. He reported being generally fit and independently active. The patient’s regular GP reviews had noted no concerns. Clinical examination showed muscle wasting bilaterally in the first digital web spaces, but no muscle tenderness was demonstrated. There were no peripheral stigmata of chronic liver disease. The admission blood test showed deranged liver functions with raised bilirubin and transaminases and acute kidney injury (AKI). Blood test results are shown in Table 1.

**Table 1:** Investigation Results

Day on admission	Day 1	Day 2	Day 3	Day 4
Sodium (mmol/L) Normal range: 133–146	131	130	128	126
Potassium (mmol/L) Normal range: 3.5–5.3	3.8	4.2	4.9	5.7
Bicarbonate (mmol/L) Normal range: 22 – 26	24	22	20	19
Urea (mmol/L) Normal range: 2.5 – 7.8	21.6	27.1	32.1	37.9
Creatinine (µmol/L) Normal range: 59–104	159	253	395	478
Alanine Transaminase (U/L) Normal range: <41	1134	976	965	947
Alkaline Phosphatase (U/L) Normal range: 30–130	200	157	148	146
Aspartate Transaminase (U/L) Normal range: 1 – 45		3062	2787	
Bilirubin (µmol/L) Normal range: <21	80	65	53	65
Creatinine Kinase (U/L) Normal range: 40–320	>26,000	>26,000	>26,000	>26,000

The autoimmune screen and thyroid function test were normal. A computerized tomography scan showed no signs of liver disease, gallstones, renal disease, or obstructive uropathy. A creatinine kinase (CK) test was added due to a suspicion of rhabdomyolysis. The CK test was returned as significantly raised, with a value of greater than 26,000 international units per liter, which is the highest reference range recordable by the local laboratory. Further history was obtained, and investigations were undertaken, but no other cause of rhabdomyolysis was found apart from atorvastatin. Based on our current search of the literature at the time of writing this report, this is the longest interval recorded of statin-induced rhabdomyolysis with severe AKI occurring after 10 years of commencing therapy with no evidence of other hepatotoxins or change in dose of treatment. A full liver panel was ordered, including an infectious and autoimmune screen. These were unremarkable, and the thyroid function test was normal. Atorvastatin and indapamide were suspended given the raised transaminases and AKI. The patient remained anuric, and serum urea and creatine levels worsened from 21.6 mmol/L and 159 µmol/L on admission to 37.9 mmol/L and 478 µmol/L, respectively. His kidney functions did not improve with medical therapy alone, and he was referred to a tertiary hospital for renal replacement therapy on advice from the renal team.

Follow-up

Our patient was referred to a regional tertiary center for specialist renal management. Over there, in addition to ongoing treatment with intravenous fluids, including sodium bicarbonate, he had a session of hemodialysis, but this did not significantly improve his clinical status. He remained anuric, and signs of decompensated renal failure, including hyperkalemia, metabolic acidosis, and pulmonary edema, occurred. A second session of dialysis was terminated as the patient became unwell 30 minutes into the session with a low Glasgow Coma Score; however, the CT brain afterward showed no intracranial pathology. Unfortunately, he developed an opportunistic infection with hospital-acquired pneumonia, requiring supplemental oxygen. Fractional-inspired oxygen (FiO2) was titrated up to 60% to maintain a target oxygen saturation of 94-98%. He had new-onset frank bleeding per rectum, delirium with overall general decline, and became unfit for further dialysis. He received appropriate antibiotics as per hospital microbiology guidelines; the bleeding was managed as per the hospital hemorrhage protocol, but the patient continued to deteriorate clinically despite input from the renal team, the general medicine team, and the critical care team. Following a multidisciplinary team discussion with his family, our patient was commenced on end-of-life care and passed away shortly afterward.

## Discussion

Rhabdomyolysis describes the breakdown of skeletal muscles with the release of intracellular contents into the bloodstream and extracellular space, which includes myoglobin, creatinine kinase (CK), aldolase, lactate dehydrogenase, and electrolytes [3,5,6]. The etiology of rhabdomyolysis ranges from traumatic or physical to non-traumatic or non-physical causes. Traumatic or physical causes include polytrauma, motor vehicle accidents, earthquakes, prolonged immobilization due to coma, intoxication with alcohol and opiates, physical abuse, fractures, fire accidents and explosions, high-voltage electric shock, and strenuous muscular exercises. Non-traumatic or non-physical causes include medications such as alcohol, statins, colchicine, corticosteroids, amphetamines, infections such as septic shock, mycoplasma, legionella, malaria, electrolyte abnormalities such as hypokalemia, hypophosphatemia, hyperosmolar conditions, hypo and hypercalcemia, and severe dehydration, myopathies, insect bites, snake venom, autoimmune myositis, hemoglobinopathy, dysregulated body temperature, capillary leak syndrome, and drug withdrawal-like baclofen [8]. The presentation of rhabdomyolysis can range from an asymptomatic illness with elevation in the CK level to a life-threatening condition with extreme elevations in CK, electrolyte imbalances, acute kidney injury (AKI), and disseminated intravascular coagulation [3]. Clinically, rhabdomyolysis presents a triad of myalgia, weakness, and myoglobinuria, classically described as tea-colored urine [6]. Statin-induced rhabdomyolysis is a rare but potentially life-threatening complication of statin therapy [3-7]. Several risk factors that may predispose a person to develop statin-induced rhabdomyolysis include frailty, low body mass index, older age, female sex, hypothyroidism, hypertension, polypharmacy, and alcohol or drug abuse [7]. Of these, our patient had a history of hypertension and alcohol abuse. The pathogenesis of rhabdomyolysis involves a combination of direct muscle toxicity and impairment of mitochondrial function [3]. The majority of cases occur within the first few months of statin initiation or following dose escalation [8]. However, cases of delayed-onset rhabdomyolysis, similar to our patient, have been reported [6]. Patients usually present with non-specific symptoms, such as fatigue and muscle pain [3,5,6]. A review of case reports on statin-induced rhabdomyolysis noted that 60% of the patients presented with fatigue and muscle pain [8]. Our patient presented with similar symptoms. However, this is non-specific and associated with many other clinical conditions found in acute medical admissions. This patient had neither muscle tenderness nor an objective reduction in power. He reported a history of heavy alcohol use (half a bottle of whiskey a day), which could explain the raised transaminase levels. However, the absence of the stigmata of liver disease, a normal liver on bedside examination, and radiologic investigation made the assessing clinician evaluate for rhabdomyolysis even though the patient had tolerated statins for approximately 10 years. In this case, the patient's heavy alcohol intake and liver function abnormalities initially masked the diagnosis of statin-induced renal dysfunction. It is important to note that alcohol consumption can also potentiate statin-induced hepatotoxicity and myopathy [7]. Heavy alcohol intake, declining liver function, and intercurrent illness probably precipitated statin-induced rhabdomyolysis in this patient despite tolerating the dose for many years.

The management of statin-induced rhabdomyolysis includes immediate discontinuation of statin therapy, aggressive hydration to prevent AKI, and monitoring for complications, such as compartment syndrome. Renal function and electrolytes should be closely monitored, and urine output should be maintained at a sufficient level. A multidisciplinary team approach involving acute medicine, intensive medicine, and renal medicine should be considered early in the management of patients with severe rhabdomyolysis, as mortality has been reported to occur in up to 15% of cases. This case highlights the high mortality risk associated with rhabdomyolysis with severe AKI. Hemodialysis can be beneficial in patients who do not respond to initial management with intravenous fluid, electrolyte correction, and alkalinization of urine and who also have indications for hemodialysis like electrolyte abnormalities, anuria, and pulmonary edema like our patient [8,9]. However, it is generally considered that hemodialysis does not remove the myoglobin from renal tubules due to the size of the protein; hence, renal impairment may persist in spite of hemodialysis [6,8,9]. Given the high mortality risk of up to 15%, early hemodialysis may have mortality benefits in patients who have indications for renal replacement therapy [6,9].

## Conclusions

This case report highlights the high mortality risk associated with severe rhabdomyolysis secondary to statin therapy. It is a serious complication that can be delayed up to 10 years of statin use. This can cause significant renal impairment that is resistant to medical therapy, with a consequent high mortality risk despite hemodialysis. Hence a high index of suspicion is required for proper management, liaison with the renal specialist, and escalation of care. Factors like high alcohol intake can confound interpretation of results in the presence of rhabdomyolysis, which can masquerade this condition as liver disease due to raised transaminase levels.

## References

[1] (2023). WHO: Cardiovascular diseases fact sheet. WHO.

[2] (2023). NICE Clinical Knowledge Summary: CVD risk assessment and management. cited.

[3] Thompson PD, Panza G, Zaleski A, Taylor B (2016). Statin-associated side effects. J Am Coll Cardiol (Internet.

[4] Sharma U (20233). Statin-induced delayed rhabdomyolysis. BMJ Case Rep (Internet). 2019 (cited.

[5] Khan Z, Ibekwe M, Abumedian M, Yousif Y, Mlawa G (2022). A rare presentation of acute renal failure secondary to rhabdomyolysis in a patient due to atorvastatin requiring short-term renal replacement therapy. Cureus.

[6] Dalugama C, Pathirage M, Kularatne SAM (201820233). Delayed presentation of severe rhabdomyolysis leading to acute kidney injury following atorvastatin-gemfibrozil combination therapy: a case report. J Med Case Rep (Internet.

[7] (2023). British National Formulary (BNF): Atorvastatin interactions. cited.

[8] Mendes P, Robles PG, Mathur S (2014). Statin-induced rhabdomyolysis: a comprehensive review of case reports. Physiother Can.

[9] Stanley M, Chippa V, Aeddula NR (2023). Rhabdomyolysis. StatPearls Publishing.

